# Does kV Image Guidance for Bone Metastases Improve Pain Control?

**DOI:** 10.3389/fonc.2021.627282

**Published:** 2021-06-17

**Authors:** Ahmet S. Tunceroglu, Bin Gui, Shou-En Lu, Julian Sison, Rahul Parikh, Sharad Goyal, Bruce G. Haffty, Sung Kim, Salma K. Jabbour

**Affiliations:** ^1^ Department of Radiation Oncology, Metro Health Cancer Center, University of Michigan, Wyoming, MI, United States; ^2^ Rutgers Cancer Institute of New Jersey, Rutgers Robert Wood Johnson Medical School, Rutgers University, New Brunswick, NJ, United States; ^3^ Rutgers School of Public Health, Rutgers University, Piscataway, NJ, United States; ^4^ Division of Radiation Oncology, The George Washington University Medical Faculty Associates, Washington, DC, United States

**Keywords:** image guidance, palliative radiation, bone metastases, kilovoltage, IGRT (image guided radiation therapy)

## Abstract

**Purpose/Objectives:**

Despite its widespread availability, the use of kilovoltage (kV) image guidance is often related to factors such as perceived adequacy of clinical patient setup and individual practice patterns. We sought to determine whether kV image guidance in the treatment of painful bone metastases would improve therapeutic efficacy.

**Materials/Methods:**

Under an Institutional Review Board approved protocol, hospital records of 164 patients having received radiation therapy to 257 individual painful osseous metastases were retrospectively reviewed. Marginal logistic regression analyses using the generalized estimating equation (GEE) approach were used to investigate potential associations between pain reduction and several patient, disease, and treatment related variables. Correlation of kV image guidance with pain reduction was analyzed by univariate and multivariate GEE logistic regression analysis.

**Results:**

Median time to pain reduction was 3 days (range 0~109 days) from the start of radiation therapy. Pain reduction ≥ 50% was noted in 196 (77%) metastatic lesions with 136 (53%) demonstrating complete pain relief. Patients with metastatic lesions from non-small cell lung cancer experienced less pain relief (p = 0.007). Disease extension outside of bone was a negative predictor for pain reduction (p = 0.02). On univariate and multivariate logistic regression, kV image guidance demonstrated a statistically significant correlation with improved pain control in cases involving treatment of the lower extremities (p = 0.03) and those with fewer treatment fractions (p = 0.01), particularly in the setting of extra-osseous disease extension (p = 0.003).

**Conclusions:**

Kilovoltage image guidance in the treatment of painful bone metastases may offer greater pain control through improved patient setup, particularly for patients with tumors of the lower extremities, extraosseous disease extension, and fewer treatment fractions.

## Introduction

While long-term cure is the optimal outcome of radiation therapy (RT), many cancer patients will relapse and present with metastatic disease while others will be discovered to harbor widespread metastases at the initial presentation. In fact, the incidence of bone metastases have increased, believed to result largely from improved overall patient survival owing to improved cancer treatments (1, 2). Of the 1,806,590 cases of cancer estimated for 2020 in the United States, over 40% will be of cancers that commonly metastasize to the bone including breast, prostate, lung, and kidney (3). Following lung and liver, bone is the third most common site of metastatic disease (2). Using MarketScan and Medicare databases, Li and colleagues estimated the prevalence of skeletal metastases to be approximately 280,000 patients per year - a figure that the authors concluded was likely an underestimate. Indeed, other estimates are closer to 400,000 patients (4–6). Furthermore, among patients with breast and prostate cancers, the bulk of the disease at the time of death typically will be in bone (4). Among patients with non-small cell lung cancer, Kuchuk et al. reported bone metastases at diagnosis in 20-40% of patients (7).

Cancer metastases to bone lead to a myriad of sequelae, or “skeletal-related events,” for patients, their families, and the healthcare system. Pain, often intractable, is the most common clinical symptom associated with osseous metastases and the mechanisms responsible for the bone pain are poorly understood but appear to be related to osteolysis (4). This bone resorption can lead to the development of a pathologic fracture, a particularly worrisome complication, often in weight-bearing bones. Other manifestations of skeletal metastases include hypercalcemia, reduced mobility causing declines in performance status, neurologic deficits arising from nerve-compression syndromes such as spinal cord compression, reduced quality of life [i.e. reduced functional well-being, physical well-being, and emotional well-being (8, 9)], and death (2, 4, 10, 11). The effect of pathologic fractures on mortality is particularly troubling, as reports have suggested a 32% increased risk of death following non-vertebral fractures, and vertebral fractures have been associated with a 23-90% increased risk of mortality (12, 13).

Radiation therapy is a longstanding source of relief for painful bone metastases. As with any external beam radiation treatment, accurate and reproducible patient positioning is vital. Image-guided radiation therapy (IGRT) is among the available tools for achieving this accuracy, and serves an important role in modern RT, particularly in cases treated with curative intent. However, despite the widespread availability of IGRT techniques such as orthogonal kilovoltage (kV) imaging, their use often depends on factors such as individual practitioner preferences, institutional practices, the perceived adequacy of clinical patient set up and prior authorization by insurance companies. We, therefore, sought to determine whether or not daily kV image guidance provided a therapeutic benefit in the palliative treatment of osseous metastases and which patient and disease related parameters predicted for this benefit. We hypothesized that a clinically relevant benefit of kV image guidance would be realized in cases presenting with extraosseous disease extension and in those involving treatment of sites that may be more difficult to reproducibly immobilize, such as the extremities.

## Materials and Methods

### Ethics

The studies involving patient data were reviewed and approved by the Rutgers University Ethics and Compliance Committee.

### Patient Selection

Under an Institutional Review Board-approved protocol, hospital records of 255 patients who received RT for bone metastases were retrospectively reviewed. All patients were treated between 2004 - 2015 at the Rutgers Cancer Institute of New Jersey and Rutgers Robert Wood Johnson University Hospital. Ninety-one patients were excluded because of the lack of recorded pain scores, incomplete RT records, inadequate post-treatment follow up, or death within 30 days of treatment completion due to progressive systemic disease. The final cohort consisted of 164 patients having received RT. Patient self-reported pre- and post-treatment pain scores were obtained for 257 individual osseous metastases.

### Data Collection

Standard baseline evaluation included a complete medical history and physical, radiologic, and pathologic assessments. Data extracted from the charts included patient age, ethnicity, use of systemic therapy, histology, site of metastasis, prior surgical resection, extraosseous extension, and radiation treatment variables such as total dose, number of fractions, dose per fraction, and kV imaging. Patients treated with SBRT were excluded from the analysis. To gather information regarding pain response and time to response, charts were reviewed for pain at the following time points: the initial patient consultation for RT, the first on‐treatment visit, halfway through RT, at the last on treatment visit during RT, and 4 weeks after RT completion at the first follow‐up. Patients were asked to quantify their pain on the Numeric Pain Rating Scale ranging from 0-10, with 0 representing no pain and 10 indicating severe pain. This pain scale is widely utilized and has been validated as an easily applicable, sensitive and reproducible means of assessing pain (14).

At our institution, it is customary for all patients to undergo computed tomography (CT) simulation in order to obtain three-dimensional anatomy to help define treatment fields and to develop a patient-specific treatment plan. Patient set up during simulation is performed according to institutional standards, with use of immobilization devices such as knee/foot locks as needed. After CT scan acquisition, images were transferred to the Varian Eclipse^™^ Treatment Planning System (TPS, version 7.3.10; Varian Medical Systems, Palo Alto, CA) where target delineation, isocenter placement, beam placement and treatment planning were performed. Whether or not the osseous gross tumor volume (GTV) was contoured and utilization of kV imaging during treatment delivery were based on provider preference. All patients for whom conformal treatment planning was used also received megavoltage (MV) portal imaging on the first day of treatment delivery and every five fractions.

### Statistical Analyses

Reduction in patient self-reported pain scores following RT were analyzed in the context of the aforementioned patient, disease, and treatment related parameters. Pain reduction for each osseous metastasis, measured by comparing pre- and post-treatment Numeric Pain Rating Scale scores, was dichotomized by the median in the sample (i.e., ∆Pain ≤ 5 vs. ∆Pain > 5) to facilitate statistical data analysis. “Good pain response” was defined as > 5 point decrease following RT and “poor pain response” was defined as ≤ 5-point decrease. To account for potential correlation between pain scores from multiple metastases within the same patient, marginal logistic regression analyses using the generalized estimating equation (GEE) approach were performed. Specifically, bivariate analyses using GEE logistic regressions were performed to assess the association of individual factors with pain reduction. Variables demonstrating a significant association with pain relief (p < 0.10) were subsequently used to build a multivariable logistic regression model based on the back-elimination principle. Significant factors in the multivariable model were defined by p < 0.05. Because kV imaging is a variable of interest, this variable was also kept in the back-elimination procedure during creation of the multivariable model. All statistical analyses were performed using SAS University Edition (SAS Institute Inc., Cary, North Carolina).

## Results

Of the initial 255 patients identified, 91 were excluded from the study, resulting in a final cohort of 164 patients treated for 257 osseous metastases. The median patient age was 64.5 years, with 45.1% males and 54.9% females. The most common histology was breast (26.5%), followed by prostate (23.7%), and non-small cell lung (23%) cancers (NSCLC). Extraosseous disease was noted in 59.1%. The most common sites of disease were the spine (46.1%) and the lower extremities/pelvis (37.9%). Additional baseline patient characteristics are described in **Table 1**. In 51% of osseous metastases, the physician contoured the GTV. Among those contoured metastases, the median GTV was 54.2 cm^3^ (range 0.04 - 1729 cm^3^).

**Table 1:** Baseline patient characteristics.

Characteristic	Median	Range
Age (y)	64.5	30.8-87.1
GTV volume (cm^3^)	54.2	0.04-1729.0
Total fractions	10	1-30
Total dose (cGy)	2750	300-3750
Dose/fx (cGy)	300	30-1600
Pain reduction	5	-3-10
% Pain reduction	100	-300-100
% kV imaging	0	0-100
	**Frequency**	**%**
Histology		
Prostate	61	23.74
Breast	68	26.46
NSCLC	59	22.96
Small cell lung cancer	3	1.17
Renal	23	8.95
Bladder	5	1.95
Esophagus/Gastric	4	1.56
Melanoma	9	3.5
Colorectal	8	3.11
Sarcoma	5	1.95
Myeloma/Lymphoma	3	1.17
HCC	1	0.39
H&N	4	1.56
Gyn	2	0.78
Gender		
Female	141	54.86
Male	116	45.14
ECOG Performance Status		
0	82	38.3
1	69	32.2
2	49	22.9
3	13	6.1
4	1	0.5
Post-operative RT		
Yes	25	9.73
No	232	90.27
GTV contoured		
Yes	131	50.97
No	126	49.03
Extension beyond bone		
Yes	152	59.14
No	105	40.86
Disease site		
Spine	118	46.09
Upper extremities	22	8.59
Lower extremities/pelvis	97	37.89
Ribs/chest wall	18	7.03

A total of 46% of patients underwent at least one kV image during RT. Of the patients that underwent kV imaging, the mean number of fractions for which kV image guidance was utilized was 80% ± 26.5%, the median was 90%, and the first and third quartiles were 70% and 100%, respectively. The median total dose was 2750 cGy (range 300~3750 cGy) delivered over 10 fractions (range 1~17). The median of baseline reported pain score was 8 (range 1~10). For patients receiving 1-5 fractions, the median pain reduction was 5 (range -3~10) with a median interval of 2 days (range 0~94 days) from the start of treatment, while for patients receiving 6-10 fractions, the median pain reduction was 6 (range 0~10) with a median interval of 10 days (range 0~109 days) from the start of treatment.

92.6% (n = 238/257) of cases had any pain relief, 76.2% (n = 196/257) had ≥ 50% pain relief, and 52.9% metastases (n = 136/257) achieved complete pain relief. One hundred metastases (38.9%) received pain relief during treatment.

### Histology

There was noted to be a significant difference in pain reduction among different types of cancer (**Table 2**). Specifically, pain due to metastatic lesions of NSCLC-origin was possibly less likely to demonstrate good response, compared to pain due to metastatic lesions of non-NSCLC origin (p = 0.007).

**Table 2:** Differences in pain reduction according to parameters investigated.

Parameter	Absolute pain reduction	P value
Histology		0.007
NSCLC	4 (-3 ~10)	
Other	5 (-2 ~10)	
Age		0.94
< 65	5 (-3 ~10)	
≥ 65	5 (-3 ~10)	
Gender		0.21
Male	5 (-3 ~10)	
Female	5 (-2 ~10)	
ECOG Performance Status		0.55
0	5 (-3 ~10)	
1	6 (-3 ~10)	
2	5 (-2 ~10)	
3	4.5 (0 ~10)	
4	9 (9~9)	
Postoperative		0.74
Yes	4.5 (2 ~10)	
No	5 (-3 ~10)	
Extension beyond bone		0.23
Yes	5 (-1 ~10)	
No	6 (-3 ~10)	
Site		0.85
Spine	5 (-3 ~10)	
Upper extremities	5 (1 ~10)	
Lower extremities/pelvis	5 (-3 ~10)	
Ribs/chest wall	5 (0 ~10)	
Other	2 (2 ~2)	
Total dose		0.23
≥2800	5 (-3 ~10)	
<2800	5 (-2 ~10)	
Dose/fx		0.26
>200 cGy	5 (-3 ~10)	
≤200 cGy	8 (1 ~10)	
Dose/fx		0.17
>300 cGy	5 (-3 ~10)	
≤300 cGy	5 (-2 ~10)	
Number of fractions		0.06
1	3 (-3~10)	
>1	5 (-2~10)	
% kV imaging		0.07
<90	5 (-3 ~10)	
≥90	6 (0 ~10)	
GTV contoured (entire cohort)		0.54
Yes	5 (-2 ~10)	
No	5 (-3 ~10)	
GTV contoured (patients with extraosseous disease extension)		0.55
Yes	5 (-1~10)	
No	5 (0 ~10)	
GTV volume (n = 131)		0.96
≥55 cm^3^	5 (-2 ~10)	
<55 cm^3^	5 (-1 ~10)	

### Extraosseous Disease Extension

Though a difference in absolute pain reduction was not detected between metastatic lesions with and without extraosseous extension (p = 0.23) (**Table 2**), we did find a statistically significant difference when dichotomizing by pain relief of ∆5 points (p = 0.03) (**Table 3**). Similarly, extension beyond bone was found to be the only significant factor negatively predicting for pain relief on multivariate regression analysis, with an odds ratio of 0.58 for ∆5-point pain reduction (p = 0.02), suggesting less effective pain control among patients with extraosseous disease (**Table 3**). There were no differences in dose, number of fractions or dose per fraction between metastatic lesions with or without extension beyond bone (p=0.37, 0.91, 0.99, respectively).

**Table 3:** Odds ratio for ∆5-point pain reduction.

Parameter	Bivariate analysis			Multivariable analysis based on backward elimination procedure		
	Odds Ratio	95% CI	P value	Odds Ratio	95% CI	P value
% kV imaging	0.84	0.51-1.39	0.08	–	–	0.09
Extension beyond bone	0.58	0.35-0.95	**0.03**	0.58	0.35-0.95	**0.02**

The bold values denote p values indicating statistical significance, i.e. p ≤ 0.05.

### kV Imaging

For the entire patient cohort, utilization of kV image guidance for ≥ 90% of the delivered fractions did not yield a statistically significant improvement in pain reduction (p = 0.07). There was, however, a statistically significant improvement in pain response with ≥ 90% kV imaging for treatments delivered to the extremities, with even greater significance for the lower extremities (p = 0.05 and p = 0.03, respectively) (**Table 4**).

**Table 4:** Effect of kV imaging on pain reduction by fraction number, disease site, and extraosseous disease extension.

	Median of Absolute Pain Reduction		
	kV images <90%	kV images ≥90%	p	
All patients (n = 188 vs 67)	5	6	0.07	
All patients with total fraction > 1 (n = 171 vs 58)	5	6	0.07	
Site = Spine (n = 86 vs 24)	5	5.5	0.86	
Site = Lower extremities (n = 59 vs 26)	5	6.5	**0.03**	
Site = Upper and lower extremities (n = 73 vs 31)	5	6	**0.05**	
Total fractions = 5 (n = 57 vs 24)	5	6.5	**0.01**	
Total fractions ≤5 (n = 78 vs 38)	4	5.5	**0.05**	
Total fractions >5 (n = 110 vs 31)	5	6	0.64	
Total fractions = 10 (n = 77 vs 22)	5	6	0.74	
Total fractions ≤ 10 (n = 165 vs 61)	5	6	0.19	
Total fractions >10 (n = 23 vs 8)	5	7	0.41	
Patients with extraosseous disease (n = 112 vs 39)	5	5.5	0.37	
Site = Spine (n = 58 vs 20)	5	5.5	0.42	
Site = Lower extremities (n = 33 vs 14)	5	5	0.08	
Site = Upper and lower extremities (n = 42 vs 18)	5	5	0.10	
Total fractions = 5 (n = 34 vs 14)	4	7	**0.003**	
Total fractions = 10 (n = 44 vs 12)	5	6.5	0.86	
Patients with total fractions = 1 (n = 13 vs 13)	2	4	0.35	

The bold values denote p values indicating statistical significance, i.e. p ≤ 0.05.

The benefit of kV imaging also appeared to be dependent on fractionation insofar that 10 fraction regimens appeared to derive no statistically significant benefit from kV imaging (p = 0.74) in contrast to 5-fraction regimens (p = 0.01) (**Table 4**). The greater benefit of kV imaging in 5-fraction as opposed to 10 fraction treatment courses was more pronounced for cases with extraosseous disease extension, p = 0.86 for 10 fractions and p = 0.003 for 5 fractions (**Table 4**). There were no statistically significant differences between patients receiving kV imaging for < 90% vs ≥ 90% of fractions with respect to age, total radiation dose delivered, histology, gender, extraosseous extension, or disease site (**Table 5**).

**Table 5:** Baseline patient characteristics with respect to kV image use.

	< 90% kV imaging	≥ 90% kV imaging	P value
	Median		
Age (y)	64.4	64.4	0.92
Total dose (cGy)	3000	2000	0.17
	**Frequency**		
Histology			0.07
Prostate	50	11	
			
Breast	42	26	
NSCLC	45	14	
Small cell lung cancer	1	2	
Renal	19	4	
Bladder	4	1	
Esophagus/Gastric	2	2	
Melanoma	9	0	
Colorectal	3	5	
Sarcoma	4	1	
Myeloma/Lymphoma	2	1	
HCC	1	0	
H&N	3	1	
Gyn	2	0	
Gender			0.15
Female	98	43	
Male	90	26	
Extension beyond bone			0.82
Yes	112	40	
No	76	29	
Disease site			0.98
Spine	87	31	
Upper extremities	16	6	
Lower extremities/pelvis	70	27	
Ribs/chest wall	13	5	

### Fraction Number

The only condition in which we detected an effect of fractionation was among patients who received kV imaging for < 90% of fractions - in these patients, regimens consisting of ≤ 5 fractions yielded less pain relief than those consisting of > 5 fractions (p = 0.03) (**Table 6**).

**Table 6:** Effect on pain relief of fractionation with respect to kV image use.

	Median of Absolute Pain Reduction					
	kV images <90%		p	kV images ≥90%		p
All patients (fraction ≤ 5 vs >5)	4	5	**0.03**	5.5	6	0.96
All patients (fraction ≤10 vs >10)	5	5	0.36	6	7	0.18

The bold values denote p values indicating statistical significance, i.e. p ≤ 0.05.

### Time to Pain Reduction

We hypothesized that in addition to overall pain reduction, the time interval between the start of palliative radiation and the realization of pain reduction may also be correlated with kV imaging and fractionation, with faster pain relief through increased kV image guidance and hypofractionation, particularly for sites that may be more difficult to accurately set up on a daily basis, such as the spine or extremities. However, we were unable to detect any association between pain relief and kV image use or fractionation for any particular disease site or for patients with extraosseous disease spread (**Table 7**).

**Table 7:** Effect of kV imaging, fraction number and fraction size on time to pain relief.

	kV imaging p values	Fractionation p values	200 cGy/fx p values	300 cGy/fx p values
All patients	0.19	0.22	0.95	0.95
Site = Spine	0.44	0.53	0.87	0.41
Site = Lower extremities	0.20	0.63	0.94	0.56
Site = Upper & lower extremities	0.32	0.75	0.96	0.83
Patients with extraosseous disease	0.86	0.28	0.80	0.68

### GTV Contouring

While some physicians routinely contour the GTV for palliative cases, this practice is not universal. We analyzed whether or not contouring of the target volume improved pain reduction both in the entire patient cohort, and as well as among patients with extraosseous disease extension. However, we did not detect evidence of greater pain relief with target volume contouring in either patient population (**Table 2**).

## Discussion

We report the findings of our analysis, which did detect a correlation between improved pain relief and kV imaging. As hypothesized, the benefit of kV imaging was realized most in cases involving treatment of the lower extremities, which might be expected to be difficult to reproducibly immobilize on a daily basis. We also report improved pain control with kV imaging, particularly in cases with fewer fractions (5 vs 10 fractions). This finding is not unexpected in that regimens with fewer total fractions would conceivably provide less opportunity to “compensate” for fractions with inaccurate positioning. We also report that pain due to metastatic lesions from NSCLC may be less likely to respond to RT as compared to other types of cancer.

Our study addresses the paucity of data with regard to the benefit of routine kV image guidance during conventionally fractionated RT, specifically in the setting of palliative radiotherapy for skeletal metastases. We report that RT remains a highly effective means of palliation for pain resulting from osseous metastases, with 93% of patients reporting pain reduction and 53% reporting complete pain relief. This is consistent with reports in the literature of complete pain relief ranging from 15 to 54% and partial pain relief ranging from 28 to 89% (15–20).

Notably, though the time interval between radiotherapy and pain relief is classically thought to be several weeks, we found the median time to any pain reduction to be 3 days from the start of treatment. We attempted to collect data on pain medication use; however, as this information was not consistently available and given the inherent uncertainly associated with retrospectively correlating pain scores with pain medication use, with other complicating factors such as consistency and schedule of their usage, this data was ultimately excluded from the final analysis. Though a formal statistical evaluation of pain medication use could not be completed, it is our institutional practice to maintain patients on their pretreatment pain regimen during palliative radiotherapy and, within our cohort of patients, we did not detect an overt increase in pain medication use following radiotherapy that could explain the significantly shorter time to pain relief in the current study.

Though we did not uncover a benefit of kV image guidance specifically with respect to cases with extraosseous disease extension, we did find that extraosseous disease extension was a significant negative predictor of pain reduction (Odds ratio 0.58 for > 5-point pain reduction). This result may not be entirely surprising since disease extension beyond bony confines would be expected to result in significant pain and these patients, specifically, may consistently derive benefit from palliative radiotherapy, whether or not kV image guidance is utilized, provided that adequate margins and accurate patient set up are ensured. In support of this finding, we did find that the interaction between kV imaging and pain reduction following regimens consisting of fewer fractions was most significant in cases presenting with extraosseous disease extension. Although others have investigated the time burden of IGRT in the treatment of bone metastases (21), to our knowledge, the current study is the first to address the effect of IGRT on treatment efficacy.

While some physicians routinely contour the GTV for palliative cases, this practice is not universal. Though it was expected that contouring of the target volume would improve pain reduction, we did not detect evidence of greater pain relief with target volume contouring in either the entire patient cohort or the subgroup of patients with extraosseous disease extension.

The implications of this study are manifold. Procurement of kV images during treatment is a time-consuming process that places a demand on staff and equipment while also exposing the patient to additional radiation as well as increasing the risks associated with longer time on the treatment table. However, despite these potential drawbacks of kV image guidance, our study suggests that there may be certain patients for whom the benefits outweigh the risks.

Our findings suggest that while, for the majority of cases involving osseous metastases, palliative RT may be effectively delivered without the reliance on routine kV image guidance and without compromising efficacy, select groups of patients may be better served by incorporation of kV image guidance. Alternatively, and at a minimum, awareness of these difficult cases and mitigation of inaccurate treatment delivery through careful patient setup should be encouraged - this could prove to be useful information in countries lacking facilities capable of image guidance and in countries whose limited resources are taxed by sheer patient volume.

The current report is not without its limitations. Most significantly, the retrospective nature of our study inherently places restrictions on the conclusions that may be drawn. Additionally, while the present work suggests a possible added benefit of daily kV image guidance, this was in the setting of a large academic center with experienced radiation therapists trained in the utilization of kV image guidance for accurate treatment delivery. The importance of accurate patient set up cannot be overstated. Furthermore, it is conventional to apply generous field margins in palliative cases, which would certainly obviate the need for image guidance, but comes with the inevitable consequence of radiation delivery to healthy tissue. Our study did not examine the effect of margin size on the benefit of kV image guidance, and it may be that kV imaging is not necessary after a certain margin size. It is also conceivable, however, that radiation field margins may be reduced, in order to decrease normal tissue exposure, to a point at which kV image guidance for accurate daily patient set up becomes critical for all sites, especially in cases with extraosseous disease extension. Additionally, in our analysis we chose to dichotomize the use of kV image guidance according to the proportion of fractions in which it was utilized (≥90% vs <90%). The 90% cut-off value was chosen as it was the median percentage of fractions incorporating kV imaging. This value also made it possible to evaluate patients who received image guidance for nearly every fraction while making allowances for differences in physician practices, changes in institutional processes, and the retrospective nature of our study. Future work examining the effect of margin size on the benefit of daily kV imaging, the effect of kV imaging on duration of pain relief, as well as the minimum proportion of fractions that must be delivered with kV imaging in order to improve outcomes, may aid in clarification of the role of kV image guidance in the palliative setting.

## Data Availability Statement

The raw data supporting the conclusions of this article will be made available by the authors, without undue reservation.

## Ethics Statement

The studies involving patient data were reviewed and approved by the Rutgers University Ethics and Compliance Committee.

## Author Contributions

AT is the lead author of this manuscript and performed the majority of data collection and manuscript preparation. BG, SJ and JS assisted with data collection. All authors assisted with manuscript preparation. S-EL provided statistical analysis. SJ is the corresponding author. The study was conceived by SJ. All authors contributed to the article and approved the submitted version.

## Conflict of Interest

The authors declare that the research was conducted in the absence of any commercial or financial relationships that could be construed as a potential conflict of interest.
